# Indication for endoscopic retrograde cholangiopancreatography and development of hemorrhage: a systematic review and meta-analysis

**DOI:** 10.1093/jcag/gwae014

**Published:** 2024-04-26

**Authors:** Michael K Parvizian, Mitchell V Edwards, Prem Bhoey, Melanie C Zhang, Lawrence C Hookey, David M Rodrigues

**Affiliations:** Gastrointestinal Diseases Research Unit, Department of Medicine, Queen’s University, Kingston, ON, Canada; Gastrointestinal Diseases Research Unit, Department of Medicine, Queen’s University, Kingston, ON, Canada; Gastrointestinal Diseases Research Unit, Department of Medicine, Queen’s University, Kingston, ON, Canada; Department of Medicine, McMaster University, Hamilton, ON, Canada; Gastrointestinal Diseases Research Unit, Department of Medicine, Queen’s University, Kingston, ON, Canada; Gastrointestinal Diseases Research Unit, Department of Medicine, Queen’s University, Kingston, ON, Canada

**Keywords:** haemmorhage/hemmorhage, ERCP, meta-analysis, complication

## Abstract

**Background:**

Hemorrhage is a common complication associated with endoscopic retrograde cholangiopancreatography (ERCP), usually following sphincterotomy. Studies investigating risk factors for hemorrhage including ERCP indication have been conflicting. Therefore, we conducted a systematic review and meta-analysis to characterize the association between the ERCP indication and hemorrhage.

**Methods:**

Database searches of MEDLINE, EMBASE, and CENTRAL were conducted to identify articles up to December 12, 2022. Randomized trials or observational studies of adult patients undergoing ERCP were included. Quality assessment using the Cochrane Risk of Bias 2.0 and Newcastle-Ottawa Scales for randomized trials and observational studies respectively was conducted. A random effects meta-analysis generating pooled odds ratios with 95% confidence intervals was conducted.

**Results:**

A total of 1323 articles were identified of which 26 were included with up to 25 121 subjects in each meta-analysis. Rates of sphincterotomy (median 96.1%; IQR 60.5–100), biliary stent placement (median 17.2%; IQR 10.6–34.4), antiplatelet use (median 6.0%; IQR 0–10.1), and anticoagulant use (median 1.9%; IQR 0%–3.2%) varied among included studies. No specific indication was associated with hemorrhage in the meta-analyses including cholangitis (OR 1.50; 95% CI 0.97–2.32), choledocholithiasis/biliary stone (OR 1.28; 95% CI 0.95–1.73), malignancy (OR 0.97; 95% CI 0.66–1.42), sphincter of Oddi dysfunction (OR 1.32; 95% CI 0.72–2.40), and acute pancreatitis (OR 0.81; 95% CI 0.44–1.49).

**Conclusions:**

Overall, no indication was significantly associated with increased hemorrhage following ERCP. However, given limitations in the included studies (ie, significant heterogeneity between studies), additional research to better characterize these associations is needed.

**Protocol Registration Number:**

PROSPERO (CRD42021283978).

## Introduction

Endoscopic retrograde cholangiopancreatography is a minimally invasive endoscopic procedure used in the treatment of pancreatobiliary disease.^1,2^ ERCP can lead to potential complications which may contribute to morbidity for patients.^1,2^ Notable complications of ERCP include pancreatitis, hemorrhage, perforation, and cholangitis.^1,2^ While systematic reviews of risk factors for certain complications such as pancreatitis have been conducted, other clinically relevant complications of ERCP, such as hemorrhage, have not been conducted.

ERCP-associated hemorrhage can be a significant complication that may occur in 1%–15% of patients, most commonly in those receiving sphincterotomy.^1^ Hemorrhage in these cases often resolves spontaneously but may require interventions including blood transfusion, endoscopic interventions, embolization, or surgery.^1^ A variety of risk factors including the indication for ERCP, the length of sphincterotomy, and patient comorbidities have been identified as potential risk factors for hemorrhage.^1,3^ Despite the evidence to date, risk factors related to hemorrhage with ERCP remain poorly defined, likely due to small sample sizes and heterogeneity in study design.^1,3^ Some indications for ERCP such as acute cholangitis have been identified as potential risk factors for hemorrhage; however, these too have had conflicting results reported in the literature.^1–3^

Clinicians would benefit from identifying factors that influence the risk of hemorrhage with ERCP as this would aid in guiding clinical decisions and allow for informed decision-making alongside patients and families. Therefore, we conducted a systematic review and meta-analysis to determine whether a relationship existed between the indication for ERCP and the development of associated hemorrhage.

## Methods

This review was conducted and reported according to the Preferred Reporting Items for Systematic Review and Meta-analysis guidelines.^4^ The protocol for this systematic review was registered with PROSPERO, the International Prospective Register of Systematic Reviews (CRD42021283978). This article was reported in line with PRISMA standards.^4^

### Data sources and search strategy

Database searches of MEDLINE, EMBASE, and the Cochrane Central Register of Controlled Trials (CENTRAL) databases were conducted from inception until September 4, 2021, using the key terms described in Tables S1–S3 for each database with an updated search conducted until December 12, 2022. The search strategy was designed with the help of an experienced medical librarian in collaboration with the study investigators. Search terms were chosen to capture articles reporting on risk factors associated with hemorrhage and ERCP. Hand-searches of the reference lists of published review articles identified by this search were also conducted to identify additional articles of relevance.

### Study selection

Studies that investigated risk factors for ERCP-associated hemorrhage in adults were included. Studies were included if they met our predefined inclusion criteria: (1) patient age >=18, (2) randomized controlled trials or observational studies (ie, case-control, cross-sectional, or cohort studies), and (3) investigated risk factors for ERCP-associated hemorrhage (intraprocedural and/or post-procedural). Studies were excluded if they included non-human subjects or paediatric patients, were not available in English, did not investigate risk factors for ERCP-associated hemorrhage, or used an inappropriate study method (review, case-report, case-series, or abstract). Studies were included regardless of the definition of hemorrhage used. Of the included studies, those reporting on the indication for ERCP and hemorrhage were included in this publication with studies investigating other risk factors to be explored in future publications.

Following initial piloting exercises, teams of 2 reviewers (2 of M.K.P., M.E., P.B., and M.C.Z.) independently screened titles and abstracts for inclusion into the review using the Covidence platform.^5^ Any discrepancies in article selection at this stage resulted in automatic inclusion of the article to the full-text screening stage to prevent inappropriate exclusion. Following this, any articles identified as potentially meeting inclusion criteria underwent a full-text review by 2 reviewers using the Covidence platform.^5^ All discrepancies at the full-text screening stage were resolved by consensus and a third reviewer consulted if a disagreement was not resolved by this process. If authors published multiple publications using the same cohort and risk-factors, the study with the longest follow-up period was selected. Inter-rater reliability at each screening stage for each data screening pair was assessed using Cohen’s Kappa.

### Data extraction and quality assessment

A data extraction form was developed for this review using Google Sheets. Predefined variables that were extracted included the following: author, year of publication, study design, the country the study was conducted in, patient factors (sex, baseline comorbidities including patients with surgically altered anatomy, cirrhosis, and chronic kidney disease, and patient medications including antiplatelets and anticoagulants), procedural variables (including sphincterotomy and biliary stent placement), indication for ERCP, and outcome data (including the definition of hemorrhage used, number of cases, effect estimates, variability estimates, and the factors adjusted for in the analysis). When a study reported multiple adjusted estimates of an effect, the most adjusted effect estimate was abstracted.

Teams of 2 reviewers (2 of M.K.P., M.E., P.B., and M.Z.) assessed the risk of bias and methodological quality of each included study using the Cochrane Risk of Bias 2.0 (RoB 2.0) tool for each randomized study, and the Newcastle-Ottawa Scales for non-randomized studies.^6,7^ The Newcastle-Ottawa scale scores were then converted to Agency for Healthcare Research and Quality (AHRQ) standards, categorizing the non-randomized studies as good, fair, or poor quality. As no AHRQ provided score to quality rating conversion guidance exists, commonly utilized thresholds from other reviews were utilized.^8^ Observational studies with 3 or 4 selection domain stars, 1 or 2 comparability domain stars, and 2 or 3 exposure/outcome domain stars were considered good quality. Those with 2 selection domain stars, 1 or 2 comparability domain stars, and 2 or 3 exposure domain stars were considered fair quality. Those with 0 or 1 selection domain stars, 0 comparability domain stars, or 0 or 1 exposure domain stars were considered poor quality.

### Data synthesis and analysis

All statistical analyses were undertaken using the Cochrane Review Manager 5.4.^9^ A DerSimonian and Laird inverse variance random-effects meta-analysis was conducted for each indication to generate a pooled odds ratio (OR) with 95% confidence intervals (CIs) and a *P* value < 0.05 considered statistically significant. Where possible, subgroup analyses based on geographic region, age, and sex were planned to be conducted.

To evaluate heterogeneity, the percentage of variation attributable to heterogeneity (*I*^2^) was calculated for each meta-analysis with *I*^2^ cut-offs of 25%, 50%, and 75% taken to represent low, moderate, and high degrees of heterogeneity, respectively for this review.^10^ Where 10 or more studies were available for any outcome, a funnel plot was generated to assess for publication bias using Review Manager 5.4.^9^

## Results

### Study selection

**Figure 1.**
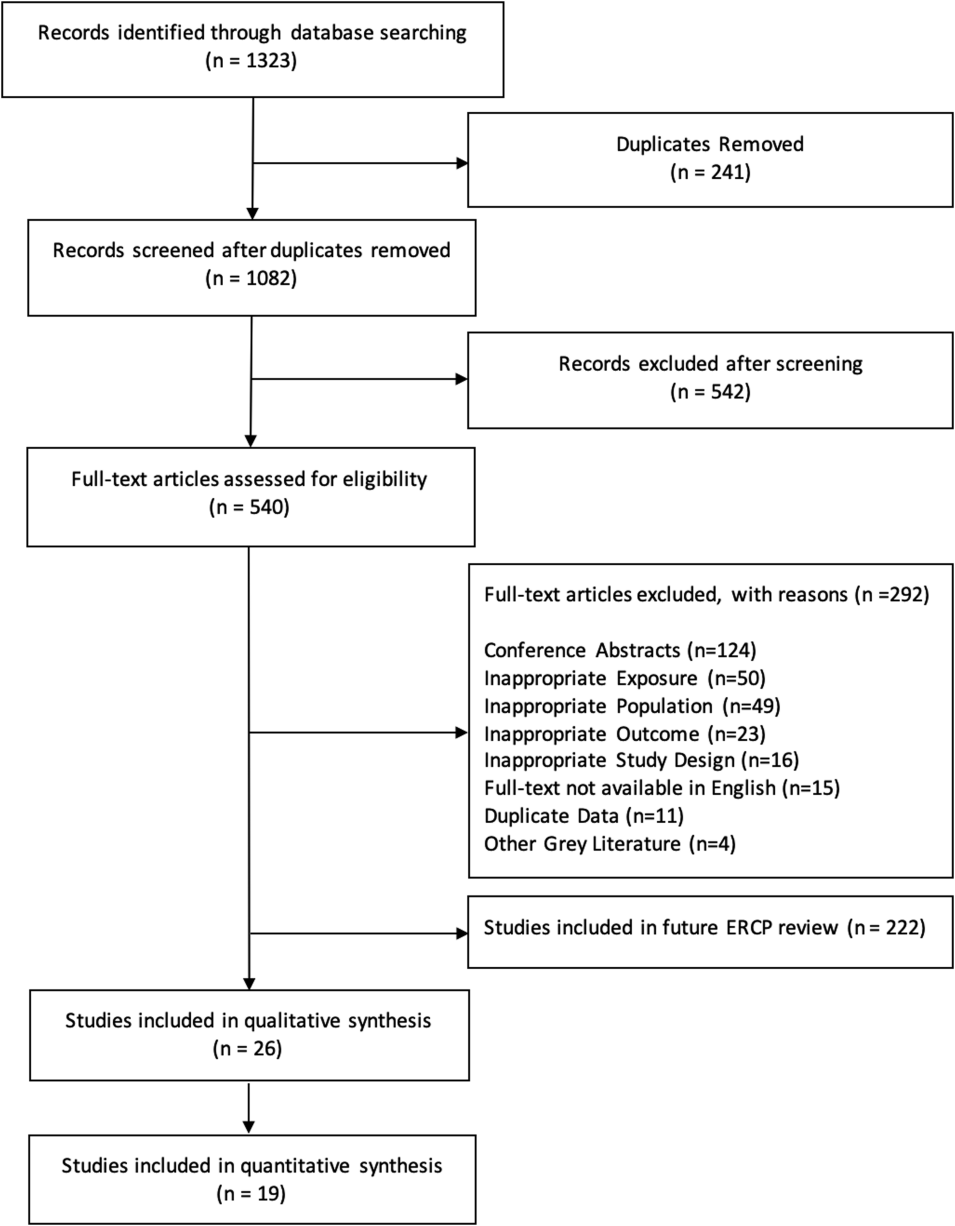
Preferred Reporting Items for Systematic Reviews and Meta-Analyses (PRISMA) diagram outlining the results of the search strategy from initial search to study inclusion.

A total of 1323 articles were identified through database and hand searches with 1082 unique articles remaining after removing duplicates. A total of 26 articles were included after screening with 19 providing sufficient data for inclusion in the meta-analysis (Figure 1).

There was a high degree of agreement between each pair of reviewers at all stages of the review with a Cohen’s Kappa ranging from 0.81 to 0.89 during the title and abstract screening and 0.80 to 0.85 for the full-text screening stage.

### Study characteristics

The majority (*n* = 24) of included studies were observational.^1–3,11–30^ The remaining 2 studies were randomized controlled trials.^31,32^ The majority of included studies were conducted in East Asia (*n* = 10), North America (*n* = 7), and Europe (*n* = 7), with only 2 studies reported from West Asia and no studies from any other geographic regions. Rates of hemorrhage differed within the included studies (*n* = 24, median 3.7%; IQR 1.6–10.0). Sphincterotomy rates in the included studies also varied (*n* = 26, median 91.9%; IQR 60.5–100) as did the rate of biliary stent placement (*n* = 8, median 17.2%; IQR 10.6–34.4). Antiplatelet (*n* = 10, median 6.0%; IQR 0–10.1) and anticoagulant (*n* = 10, median 1.9%; IQR 0%–3.2%) use was not commonly reported in the included studies. However, in the studies reporting biliary stent, antiplatelet, and anticoagulant use, the rates in relation to ERCP indication of these variables were not reported. For the studies reporting on sphincterotomy rates, the rate for each indication within the studies was not reported although 4 studies in the cholangitis, 6 in the choledocholithiasis, 4 in the malignancy, 4 in the sphincter of Oddi dysfunction (SOD), and 2 in the acute pancreatitis analyses only included patients with sphincterotomy.^1–3,14,17,21,23,25,31^ Detailed characteristics of included studies are reported in Table 1. The majority of studies included data from a single or multiple centres; however, the largest included study, which contributed the largest sample to the meta-analysis, was derived from a large population dataset that did not involve direct data capture for the sole purpose of the study.^11^ Hamada et al. reported on a sample of 61 002 patients from the Japanese Diagnosis Procedure Combination dataset, a national database that has been demonstrated to have a high degree of validity, particularly with procedural and diagnosis variables.^11,33^

**Table 1.** Characteristics of included studies

Study	Study design, country/region	Definition of hemorrhage	N	Number with hemorrhage (%)	Age (Mean, SD)	Female %	Surgically altered anatomy %	Cirrhosis %	Chronic kidney disease %	Antiplatelet use %	Anticoagulant use %	Sphincterotomy, %	Biliary stent placement %	Indication
Ak et al., 2022	Single centre retrospective cohort, Turkey	Hemorrhage occurring after sphincterotomy defined as intraprocedural bleeding if it required cushioning, sclerotherapy, electrocoagulation, and/or hemoclips with a balloon catheter. Delayed bleeding was defined as haematemesis, melena, and/or a decrease in haemoglobin of 3 g/dL after the procedure	1288	127 (10.9)^e^	61.5, 18.4	59.4	NR	NR	NR	7.9	2.2	90.3	NR	Biliary stone (88.9) Malignancy (6.5) Benign biliary stenosis (2.3) Post-transplant stricture (0.2) Other (2.0)
Bae et al., 2019	Single Centre Retrospective Cohort, South Korea	Defined as immediate if the onset of bleeding occurred during ES and delayed if the bleeding was not evident during the ES, but later manifested as melena or haematemesis, and was associated with a drop in haemoglobin levels	1121	108 (9.6)	67.9, 14.2	45.1	0	3.6	1.3	0	0	100.0	NR	Biliary stone (71.3) Stricture (8.4) Other (5.1)
Freeman et al., 1996	Multicentre Prospective Cohort, Canada/United States of America	Clinical evidence of bleeding, such as melena or haematemesis, with an associated decrease of at least 2 g per decilitre in the haemoglobin concentration, or the need for a blood transfusion	2347	48 (2.0)	60.4, 19.1	61.4	2.1	3.1	NR	NR	1.6	100.0	NR	Biliary stone (68.2) Malignancy (13.2) SOD (11.6) Pancreatitis (11.2) Stricture (4.2) Other (7.8)
Hamada et al., 2015	Multicentre Retrospective Cohort, Japan	Severe bleeding was identified based on the requirement for RBC transfusion, endoscopic hemostasis, or vascular embolization for gastrointestinal bleeding with or without the corresponding ICD-10 codes: intraperitoneal (K66.1) or gastrointestinal (K92.2) bleeding, or hemobilia (K83.8).	61002	510 (0.8)	72.3, 13.5	45.6	NR	1.2	2.4	7.3	3.2	89.3	NR	NR
Han et al., 2021	Single Centre Retrospective Cohort, United States of America	Cotton et al., 2010^c^	3023	12 (0.4)	50.3, 16.8	57.8	0	NR	NR	10.1	3.2	28.9	NR	Stricture (52.7) SOD (8.1) Pancreatitis (2.1) Tumour (1.5) Other (35.6)
Hung et al., 2019	Multicentre Retrospective Cohort, Taiwan	Endoscopic haemostatic treatment post-ERCP	3201	103 (3.2)	63.1, 13.9	29.6	NR	100	NR	NR	NR	81.8	NR	NR
Katsinelos et al., 2010	Single Centre Randomized Control Trial, Greece	Immediate bleeding was defined as a “trickle” if blood was evident, “oozing” if a perceptible blood stream was present, and “pulsatile” if arterial bleeding was evident. Delayed bleeding was defined as hemorrhage not evident at the time of sphincterotomy, but which presented subsequently as melena or haematemesis associated with a reduction in hemoglobin.	387	43 (11.1)	70.3, 13.8	55.0	0	1.8	2.6	0	NR	100.0	NR	Biliary stone (72.1) Malignancy (21.4) Cholangitis (12.1) SOD (3.4)
Katsinelos et al., 2019	Single Centre Retrospective Cohort, Greece	Bleeding was defined as evident intraprocedural bleeding, early bleeding (bleeding episode within 24 h after ES but not evident during procedure), or delayed (bleeding episode occurred up to 20 days after ES and manifested as melena, haematemesis or hematochezia associated with a decrease in haemoglobin level)	3058	97 (3.2)	64, 32.6	55.7	NR	1.0	NR	23.3	2.7	100.0	NR	Biliary stone (76.9) Malignancy (8.4) Pancreatitis (3.9) Cholangitis (1.8) Other (9.0)
Kim et al., 1999	Single Centre Prospective Cohort, South Korea	Cotton et al., 1991^c^	1304	136 (10.4)	57.3, 15.6	45.6	NR	3.5	NR	NR	NR	100.0	NR	Biliary stone (61.0) Malignancy (21.9) Stricture (3.8) SOD (0.8) Other (12.5)
Kim et al., 2010	Single Centre Retrospective Cohort, South Korea	Early hemorrhage was defined as bleeding which presented during the procedure or within 24 h after EST and significant bleeding which required endoscopic treatment for hemostasis. Delayed hemorrhage was defined as hemorrhage not evident at the end of the procedure, but which occurred 24 h after the procedure. Delayed hemorrhage was confirmed by subsequent endoscopy. Minor hemorrhage was defined as a decrease in haemoglobin of less than 2 g/dL, and major hemorrhage was defined as a decrease in haemoglobin of 2 g/dL or more, or when transfusion of more than 2 units was required.	1549	65 (4.2)	63.3, 14.3	45.9	NR	0.7	0.5	0	0	100	NR	Biliary stone (74.6) Malignancy (15.1) Papillary stenosis (7.0) Other (3.4)
Kostrzewska et al., 2011	Single Centre Retrospective Cohort, Poland	Cotton et al., 1991^d^	734	11 (1.5)	63.3, 19-99	62.2	47.1	NR	NR	NR	NR	55.0	15.4	Biliary stone (37.1) Tumour (15.4) Malignancy (10.1) Pancreatitis (8.9) Other (28.5)
Kwak et al., 2020	Multicentre Retrospective Cohort, United States of America	Cotton et al., 1991^d^	1079	18 (1.7)	<40 years (20.6%), 40-70 years (55.6%), >70 years (23.8%)	63.8	NR	NR	4.6	NR	15.6	60.5	17.7	Biliary stone (76.1) Cholangitis (11.4) Pancreatitis (9.8) Other (2.7)
Lee et al., 2014	Single Centre Retrospective Cohort, South Korea	An episode of any of the following: clinical evidence of bleeding, such as melena, haematemesis or hematochezia, or a decrease in hemoglobin of >2 g/dL from baseline after the procedure	762	79 (10.4)	63.3, 15.0	43.6	NR	NR	NR	17.3	0	100.0	NR	Biliary stone (49.5) Cholangitis (41.9)
Masci et al., 2001	Multicentre Prospective Cohort, Italy	Clinical (not just endoscopic) evidence of hemorrhage when associated with a decrease in Hb level greater than 2 g/dL.	2444	30 (1.2)	64.6, 15.7	55.5	NR	NR	NR	NR	NR	68.0	NR	Biliary stone (62.6) Malignancy (17.5) Pancreatitis (14.0) SOD (7.3)
Navaneethan et al., 2015	Single Centre Retrospective Cohort, United States of America	Cotton et al., 2010^c^	706	49 (6.9)	65.1, 16.4	47.6	0	NR	NR	NR	NR	98.7	58.8	Malignancy (47.2) Biliary stone (22.5) Stricture (12.2) Pancreatitis (11.0) SOD (4.9) Other (2.2)
Navaneethan et al., 2017	Multicentre Case-Control, United States of America	ICD-9 codes used to define post-ERCP hemorrhage (998.11, 909.3, and V58.89).	16140	NR^f^	61.3	58.5	NR	20.0	NR	NR	NR	66.4	NR	Biliary stone (75.4) Pancreatitis (32.4) Stricture (18.4) Cholangitis (15.9) Abnormal liver enzymes/jaundice (11.8)
Nelson et al., 1994	Single Centre Retrospective Cohort, United States of America	Hemoglobin drop >1 g/dL, melena, haematochezia, haematemesis, or orthostatic hypotension. Major hemorrhage was defined as clinical evidence of bleeding (melena, haematemesis, or endoscopically observed major bleeding) plus one or more of the following: hemoglobin fall >3 g/dL within 4 days, RBC transfusion, or subsequent endoscopic intervention	191	10 (5.3)	66.0, 19.0	43.0	NR	NR	NR	NR	NR	100.0	NR	Biliary stone (38.2) Cholangitis (26.7) SOD (8.9) Pancreatitis (8.4) Other (17.8)
Onal et al., 2013^a^	Single Centre Case-Control, Turkey	Hemorrhage not clearly defined. Significant hemorrhage was defined as clinical evidence of bleeding, such as melena or haematemesis, with an associated decrease of at least 2 g/dL in the hemoglobin concentration or the need for a blood transfusion	308	74 (24.0)	NR	NR	NR	NR	NR	NR	NR	13.6	5.2	NR
Rabenstein et al., 1999	Single Centre Retrospective Cohort, Germany	Decline in hemoglobin of at least 2 g/dL compared with values before ES combined with clinical signs of GI bleeding (i.e. haematemesis, tarry stool, endoscopic verification) or any need for erythrocyte transfusion.	1335	33 (2.5)	64.0, 15.0	51.8	NR	NR	NR	NR	NR	100.0	NR	Biliary stone (40.7) Malignancy (27.7) Pancreatitis (18.0) SOD (3.7) Abnormal liver enzymes jaundice (1.5) Tumour (0.3) Other (8.1)
Tanaka et al., 2015	Single Centre Randomized Trial, Japan	A bleeding complication was classified as endoscopically or clinically evident. The endoscopically evident bleeding was defined as visible during the procedure of sphincterotomy. This bleeding was temporary slight oozing and natural hemostasis was observed without requiring an endoscopic hemostatic therapy. The clinically evident bleeding was defined as per Cotton et al., 1991^d^	360	32 (8.9)	Median, Range (73, 23-101)	44.4	NR	NR	NR	NR	NR	100.0	25.8	Biliary stone (81.4) Malignancy (17.8) Pancreatitis (0.8)
Tsai et al., 2019	Multicentre Retrospective Cohort, Taiwan	Requirement for endoscopic hemostasis (order code 47043B) events appearing within 14 days after ERCP	4106	91 (2.2)	Mean, range (66.8, 33-86)	NR	NR	0	2.1	NR	NR	93.4	NR	NR
Ueki et al., 2009	Single Centre Retrospective Cohort, Japan	Any of the following conditions: appearance of tarry stool, decrease in Hb by 2 mg/dL or more, necessity of blood transfusion, and haemostatic treatments including heat probe	127	11 (8.7)	69.1, 12.2	38.6	NR	NR	NR	NR	NR	100.0	NR	Biliary stone (100.0)
Von Seth et al., 2015^b^	Multicentre Retrospective Cohort, Sweden	Intra-operative bleeding is defined as bleeding requiring intervention such as transfusion or operation with post-operative bleeding defined as patients with confirmed evidence of bleeding requiring transfusion and/or endoscopic or surgical re-intervention.	8932	89 (1.0)	NR	NR	NR	NR	NR	NR	NR	62.7	36.5	Biliary stone (37.7) Abnormal liver enzymes/jaundice (40.9) Malignancy (15.5) Primary sclerosing cholangitis (1.6) Bile leak (1.3) Other (1.3)
Wilcox et al., 2004	Single Centre Prospective Cohort, United States of America	Intraprocedural bleeding evident during endoscopy if any blood was seen with delayed hemorrhage captured and defined as per Cotton et al., 1991^d^	550	NR	54.0, 19.0	62.2	NR	NR	NR	NR	NR	100.0	NR	Biliary stone (42.9) Other (57.1)
Williams et al., 2007	Multicentre Prospective Cohort, United Kingdom	Cotton et al., 1991^d^	5264	40 (0.9)^g^	65.0, 16.7	56.8	1.3	1.4	0.3	4.6	NR	46.3	32.2	Biliary stone (54.3) Malignancy (19.5) Pancreatitis (9.5) Cholangitis (5.4) SOD (1.5) Other (19.3)
Ye et al., 2021	Single Centre Retrospective Cohort, People’s Republic of China	Immediate hemorrhage was defined as the visual observation of active bleeding or errhysis, which required the use of adrenaline, haemostatic clips, or electric coagulation to stop the bleeding and delayed hemorrhage was defined as the occurrence of postoperative haematemesis, black stool, or blood flows from the nasal biliary drainage tube, within 30 days after ERCP	1009	104 (10.3)	61.3, 14.8	44.6	4.1	3.3	NR	0	0	55.6	16.6	Biliary stone (65.3) Abnormal liver enzymes/jaundice (27.7) Stricture (23.6) Cholangitis (15.8)

NR, not reported; SD, standard deviation; SOD, sphincter of oddi dysfunction.

^a^Reported mean age and range for those with significant hemorrhage and no hemorrhage but not for all patients: significant hemorrhage 74.5 (30–79), and no hemorrhage 65.0 (18–100).

^b^Reported mean age and standard deviation, and female % for those with primary sclerosing cholangitis (PSC) and those without PSC: PSC 45 years (16) with 38% female, and non-PSC 69 years (16) with 56% female.

^c^Cotton et al. (2010) defines bleeding as hematemesis and/or melena or hemoglobin drop >2 g/L.

^d^Cotton et al. (1991) defines bleeding as mild if there is clinical evidence of bleeding with a decrease in hemoglobin level (>30 g/L) with blood transfusion not required, moderate if transfusion is required (≤4 units) but not angiographic intervention or surgery, and severe if transfusion of 5 or more units of blood or the need for surgical or angiographic intervention.

^e^125 of 1288 patients did not undergo sphincterotomy.

^f^Reported a 2.1% rate of hemorrhage in patients with cirrhosis (*n* = 3228) and 1.2% rate in those without cirrhosis (*n* = 12 912).

^g^Only recorded for patient’s first procedure. This study included 4561 unique patients who underwent a total of 5264 ERCPs.

### Indications for ERCP

Various indications for ERCP were identified, of which 5 had sufficient data for meta-analysis. These were choledocholithiasis/biliary stone, cholangitis, malignancy, SOD, and acute pancreatitis.

**Figure 2.**
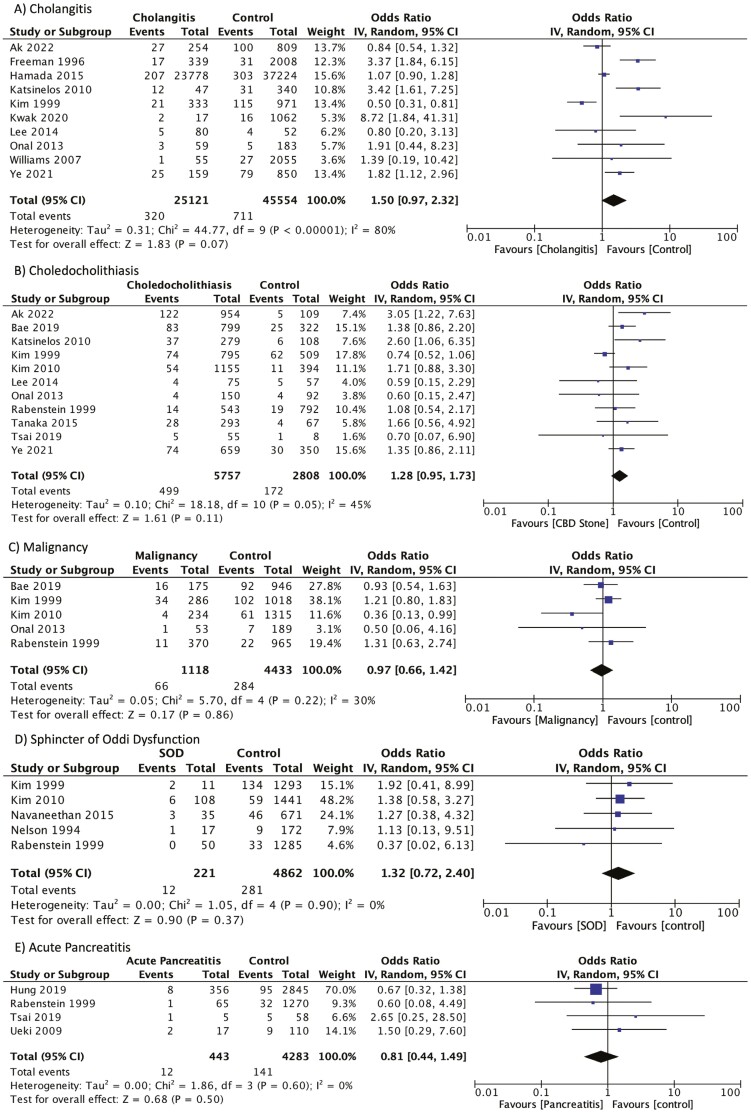
Forrest plots reporting odds of hemorrhage for each indication compared to those with all other indications: (A) cholangitis, (B) choledocholithiasis/biliary stone, (C) malignancy, (D) sphincter of oddi dysfunction, and (E) acute pancreatitis.

Ten studies were eligible for inclusion in the analysis of cholangitis, 11 for choledocholithiasis, 5 for malignancy, 5 for SOD, and 4 for acute pancreatitis.^1–3,11,12,14,16,20,22–25,27,28,30–32^ Hemorrhage was not significantly associated with any indication cholangitis (OR 1.50; 95% CI 0.97–2.32), choledocholithiasis/biliary stone (OR 1.28; 95% CI 0.95–1.73), malignancy (OR 0.97; 95% CI 0.66–1.42), SOD (OR 1.32; 95% CI 0.72–2.40), or acute pancreatitis (OR 0.81; 95% CI 0.44–1.49) during the meta-analyses (Figure 2A–E).

A high-degree of heterogeneity was noted in the meta-analysis of cholangitis (*I*^2^ = 80%) with a low-degree of heterogeneity noted in the choledocholithiasis (*I*^2^ = 45%) and malignancy (*I*^2^ = 30%) meta-analyses and no significant heterogeneity noted in the SOD (*I*^2^ = 0%) and acute pancreatitis (*I*^2^ = 0%) analyses. Of the subgroups identified *a priori*, none had sufficient studies in each group to perform a subgroup analysis.

Several other indications for ERCP were investigated but had insufficient data for meta-analysis. Chronic pancreatitis was investigated in several studies included in this review. Rabenstein et al. reported on a retrospective cohort study, detailing that 2.9% of their patients with chronic pancreatitis developed hemorrhage related to ERCP compared to an overall cohort hemorrhage incidence of 2.5%.^23^ Han et al. reported on the odds of hemorrhage in those with an ERCP indication of chronic pancreatitis (OR 0.6; 95% CI 0.2–1.9) compared to those without chronic pancreatitis and did not note any statistically significant differences.^34^ Ye et al. examined the proportion of patients experiencing no hemorrhage, immediate hemorrhage, or delayed hemorrhage.^28^ In their total sample, they reported varying rates of hemorrhage between those whose indication was jaundice (89.7%, 7.5%, and 2.8% had no, immediate, or delayed hemorrhage respectively) and those whose indication was pancreatic disease (87.5%, 7.2%, and 5.3% had no, immediate, or delayed hemorrhage, respectively).^28^ Wilcox et al. reported immediate hemorrhage and hemorrhage at 5 min after endoscopic sphincterotomy.^26^ In those whose indication was biliary dilation compared to those without, there was no significant difference in immediate hemorrhage (OR 0.78; 95% CI 0.31–1.9) or hemorrhage at 5 min (OR 0.63; 95% CI 0.2–1.6).^26^ Navaneethan et al. reported on predictors of post-ERCP hemorrhage including in those whose indication was for a suspected biliary pathology compared to a pancreatic pathology and did not find any significant differences between the groups (OR 1.5; 95% CI 0.8–2.6).^19^ Von Seth et al. reported on ERCP outcomes in those with primary sclerosing cholangitis compared to those without finding no significant difference in rates of hemorrhage between groups (0.7% vs. 1%).^29^

### Study quality and bias

**Figure 3.**
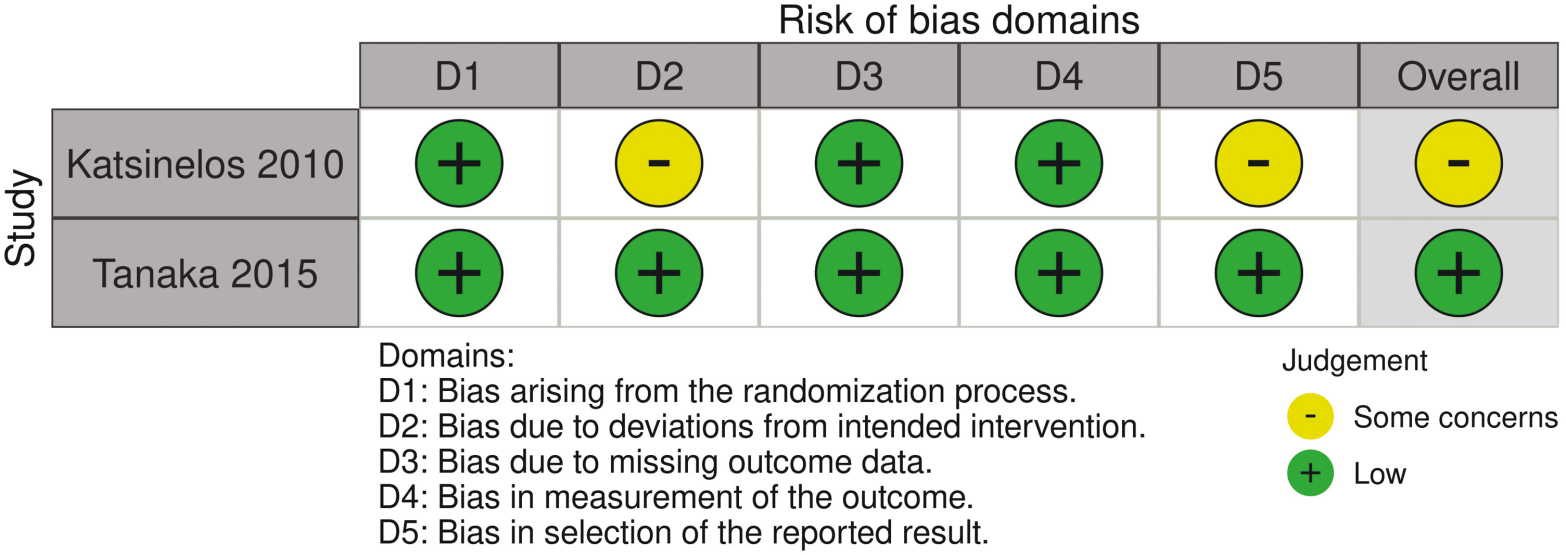
Quality assessment of randomized controlled trials

Tables 2–3 and Figure 3 present the risk of bias in the included studies. For the 22 included cohort studies (Table 2), 11 studies were considered “good” quality, 0 were considered “fair” quality, and 11 were considered “poor” quality. Of the 2 included case–control studies (Table 3), both studies were considered “fair” quality. One of the 2 randomized controlled trials was considered at “low” risk of bias whereas there were “some concerns” with the other using the Cochrane risk-of-bias tool 2.0 (Figure 3).

**Table 2.** Quality assessment of cohort studies.

	Selection				Comparability	Outcome				
Study	Representativeness of the exposed cohort	Selection of the non-exposed cohort	Ascertainment of exposure	Demonstration that the current outcome of interest was not present at the start of study	Comparability of cohorts on the basis of the design or analysis	Assessment of outcome	Was follow-up long enough for outcomes to occur	Adequacy of follow-up of cohorts	Total Score/9	AHRQ Quality rating
Ak et al., 2022	*	*	*	*	*	*			6	Poor
Bae et al., 2019	*	*	*	*	*			*	6	Poor
Freeman et al., 1996	*	*	*	*	**	*	*	*	9	Good
Hamada et al., 2015	*	*	*	*	*	*		*	7	Good
Han et al., 2021		*	*	*	**	*		*	7	Good
Hung et al., 2019		*	*	*	**	*	*	*	8	Good
Katsinelos et al., 2019	*	*	*	*	*	*		*	7	Good
Kim et al., 1999	*	*		*			*	*	5	Poor
Kim et al., 2010		*		*				*	3	Poor
Kostrzewska et al., 2011	*	*	*	*	*	*		*	7	Good
Kwak et al., 2020	*	*	*	*	**	*		*	8	Good
Lee et al., 2014	*	*	*	*		*		*	6	Poor
Masci et al., 2001	*	*	*	*	**	*		*	8	Good
Navaneethan et al., 2015	*	*	*	*	**	*		*	8	Good
Nelson et al., 1994	*	*	*	*	*	*		*	7	Good
Rabenstein et al., 1999	*	*	*	*		*		*	6	Poor
Tsai et al., 2019		*	*	*		*	*	*	6	Poor
Ueki et al., 2009		*		*	**				4	Poor
Von Seth et al., 2015		*	*	*		*	*		5	Poor
Wilcox et al., 2004	*	*	*	*	*	*	*	*	8	Good
Williams et al., 2007	*	*	*	*		*	*	*	7	Poor
Ye et al., 2021	*	*	*	*		*	*		6	Poor

Threshold for converting the Newcastle-Ottawa scale to AHRQ standards: Good quality: 3 or 4 stars in selection domain AND 1 or 2 stars in comparability domain AND 2 or 3 stars in outcome domain. Fair quality: 2 stars in selection domain AND 1 or 2 stars in comparability domain AND 2 or 3 stars in outcome domain. Poor quality: 0 or 1 star in selection domain OR 0 stars in comparability domain OR 0 or 1 stars in outcome domain.

**Table 3:** Quality assessment of case-control studies

	Selection				Comparability	Exposure				
Study	Adequate case definition	Representativeness of the cases	Selection of controls	Definition of controls	Comparability of cases and controls on the basis of the design or analysis	Ascertainment of exposure	Same method of ascertainment for cases and controls	Non-response rate	Total Score/9	AHRQ Quality rating
Navaneethan et al., 2017		*		*	**	*	*	*	7	Fair
Onal et al., 2013		*		*	**	*	*	*	7	Fair

Threshold for converting the Newcastle-Ottawa scale to AHRQ standards: Good quality: 3 or 4 stars in selection domain AND 1 or 2 stars in comparability domain AND 2 or 3 stars in exposure domain. Fair quality: 2 stars in selection domain AND 1 or 2 stars in comparability domain AND 2 or 3 stars in exposure domain. Poor quality: 0 or 1 star in selection domain OR 0 stars in comparability domain OR 0 or 1 stars in exposure domain

There were sufficient volumes of studies in the choledocholithiasis/biliary stone and cholangitis meta-analyses to generate funnel plots to assess for publication bias. Some asymmetry was noted in the funnel plots (Figure S1) with a relative paucity of studies with higher standard errors that rejected the null hypothesis in both analyses. There was a relative lack of studies suggesting increased odds of hemorrhage in the choledocholithiasis/biliary stone and decreased odds of hemorrhage in the cholangitis analysis. This suggests that the effect estimate for the choledocholithiasis/biliary stone and cholangitis meta-analyses may be underestimating the association between hemorrhage and choledocholithiasis due to missing data.

## Discussion

This systematic review and meta-analysis demonstrate that there is no apparent difference in the association of hemorrhage with the indication of ERCP, be it acute cholangitis, choledocholithiasis, malignancy, acute pancreatitis, or SOD. However, the confidence intervals were wide and there was substantial heterogeneity in several of the meta-analyses. Nonetheless, this is the first meta-analysis investigating a possible link between the indication for ERCP and hemorrhage and contributes to the current knowledge base surrounding risk factors for hemorrhage associated with ERCP in an area with conflicting results in previously conducted research.^1–3^

Hemorrhage is a common complication associated with ERCP and can result in significant morbidity for patients and costs to the healthcare system. In our study, the median rate of hemorrhage was 3.7%, highlighting the significant proportion of patients affected by this complication. One Canadian study performed in the province of Quebec by Adam et al. (2015) investigated costs associated with an ERCP-first compared to MRCP-first approach in those presenting with biliary obstruction.^35^ Of note, they investigated the cost of ERCP complications as part of their cost-analysis, demonstrating that the cost of hospitalization for gastrointestinal hemorrhage after ERCP was 3677 Canadian dollars on average which did not include physician fees or the opportunity costs for patients in terms of lost productivity in their own work due to these complications.^35^ While exact costs may vary by centre, hemorrhage has clear impacts on both a large proportion of patients and the healthcare system at large. Our review investigates multiple indications for hemorrhage as potential risk factors for this significant complication, although based on our findings we are unable to suggest that certain indications are associated with a higher risk of post-ERCP hemorrhage.

There are numerous strengths to our review methodology. We used a broad definition of hemorrhage to ensure that all potentially eligible studies were identified and included in the review. Similarly, we used an automatic inclusion method for our title and abstract screening discrepancies to ensure that in cases of disagreements between screeners, no article was inadvertently excluded without a full-text review. Additionally, we utilized broad search terms to identify as many potential articles for inclusion as possible at the initial database search stage. Together, these methods reduce the risk of missed articles in this review. We also demonstrated a high degree of inter-rater reliability at all stages of the review, suggesting that our screening tool and process were consistently applied between reviewers. Additionally, we extracted and assessed the quality of all articles in duplicate to ensure the accuracy of collected data. Lastly, by including all identified ERCP indications we were able to contextualize our findings for each indication by providing relative odds of hemorrhage across a range of presentations which provides a more detailed view of the impact of each indication for ERCP on hemorrhage.

There were several key limitations to this review. Firstly, we were unable to distinguish between post-procedural and intraprocedural hemorrhage in our meta-analysis, both of which may have different risk-factors. Another limitation includes the varying degrees of heterogeneity in our meta-analyses, which is potentially indicative of publication bias, varying definitions of hemorrhage, or other differences in study design and populations. For the cholangitis meta-analysis in particular, the high degree of heterogeneity would suggest that the pooled result may not represent the true association. Specifically, the funnel plots for the choledocholithiasis/biliary stone and cholangitis meta-analyses suggested potential missing studies supportive of increased and decreased odds of hemorrhage with these indications respectively. Furthermore, while there are multiple studies using similar definitions of hemorrhage such as those defined by Cotton et al. in 1991 or 2010 (*n* = 7), the majority used a variety of other definitions.^36,37^ While the decision to include all definitions was made for pragmatic reasons recognizing the varied criteria to define hemorrhage used by authors this does introduce a potential source of heterogeneity and may explain some of the heterogeneity in the choledocholithiasis and cholangitis meta-analyses. Additionally, in the choledocholithiasis meta-analysis which also had notable heterogeneity, the majority of studies did not delineate cholangitis as a specific indication^14,22,30,31^ (*n* = 4) or would include those with concurrent cholangitis^17,28^ (*n* = 2), with only one study clearly separating these indications in their reporting^23^ and the rest not reporting on whether patients with cholangitis were included in their studies at all (*n* = 4).^1,3,24,32^ This may have introduced some of the heterogeneity found with this analysis. The lack of data in the included studies surrounding sphincterotomy, cirrhosis, antiplatelet, and anticoagulant use stratified by the different indications further raises concern that important confounders may not have been balanced between indications in the included studies. This is important as the ability to conduct a meta-analysis stratified by key risk factors such as anti-thrombotic use was not possible but would be valuable information for patients and healthcare providers. Lastly, an important limitation of our study is the method by which each indication was compared to other groups. Each indication was compared against all other patients without that indication thus creating a heterogenous group as the comparator for each analysis. While this method was chosen due to the relative lack of data comparing specific indications to each other, it does raise concerns that important between-group differences may not be well captured by this review.

Hemorrhage is an important complication of ERCP. Our findings suggest that there are no significant differences between indications for ERCP and odds of hemorrhage, however important limitations exist. In particular, the inability to stratify results by important potential contributing risk factors (ex. sphincterotomy, anti-thrombotic use, etc.) as well as heterogeneity in the cholangitis and choledocholithiasis meta-analyses are key knowledge gaps. In the future, efforts such as the Calgary Registry for Advanced and Therapeutic Endoscopy which collects data on a large number of ERCPs with consistent definitions of outcomes and the ability to account for key confounders will be important to advance our understanding of the risk factors for complications such as hemorrhage in ERCP.^38^

## Supplementary data

Supplementary data are available at *Journal of the Canadian Association of Gastroenterology* online.

gwae014_suppl_Supplementary_Tables

gwae014_suppl_Supplementary_Figure_S1

gwae014_suppl_Supplementary_Materials

## Data Availability

Data available on request
