# Supplementary material for: Indication for endoscopic retrograde cholangiopancreatography and development of hemorrhage: a systematic review and meta-analysis
Source: J Can Assoc Gastroenterol. 2024 Apr 26;7(5):352–67. doi: 10.1093/jcag/gwae014 (PMC11477979; doi:10.1093/jcag/gwae014)

**SUPPLEMENTARY APPENDIX**

**Supplementary Table S1:** Search strategy in MEDLINE

| # | Search Terms |
| --- | --- |
| 1 | exp Cholangiopancreatography, Endoscopic Retrograde/ |
| 2 | endoscopic retrograde cholangiopancreatography.mp. |
| 3 | ERCP.mp. |
| 4 | Exp Hemorrhage/ |
| 5 | Bleed*.mp |
| 6 | Hemorrhag*.mp |
| 7 | Risk Factor*.mp |
| 8 | Exp Risk Factors/ |
| 9 | 1 or 2 or 3 |
| 10 | 4 or 5 or 6 |
| 11 | 7 or 8 |
| 12 | 4 and 8 and 11 |

**Supplementary Table S2:** Search strategy in EMBASE

| # | Search Terms |
| --- | --- |
| 1 | exp endoscopic retrograde cholangiopancreatography/ |
| 2 | endoscopic retrograde cholangiopancreatography.mp. |
| 3 | ERCP.mp. |
| 4 | exp bleeding/ |
| 5 | bleed*.mp. |
| 6 | hemorrhag*.mp. |
| 7 | exp risk factor/ |
| 8 | Risk Factor*.mp. |
| 9 | 1 or 2 or 3 |
| 10 | 4 or 5 or 6 |
| 11 | 7 or 8 |
| 12 | 9 and 10 and 11 |

**Supplementary Table S3:** Search strategy in CENTRAL

| # | Search Terms |
| --- | --- |
| 1 | exp Cholangiopancreatography, Endoscopic Retrograde/ |
| 2 | endoscopic retrograde cholangiopancreatography.mp. |
| 3 | ERCP.mp. |
| 4 | Exp Hemorrhage/ |
| 5 | Bleed*.mp |
| 6 | Hemorrhag*.mp |
| 7 | Risk Factor*.mp |
| 8 | Exp Risk Factors/ |
| 9 | 1 or 2 or 3 |
| 10 | 4 or 5 or 6 |
| 11 | 7 or 8 |
| 12 | 4 and 8 and 11 |

**Supplementary Figure S1:** Funnel plot assessing publication bias in the meta-analysis of indications and ERCP-related hemorrhage: A) Choledocholithiasis/ Biliary stone, B) Cholangitis

1. Choledocholithiasis


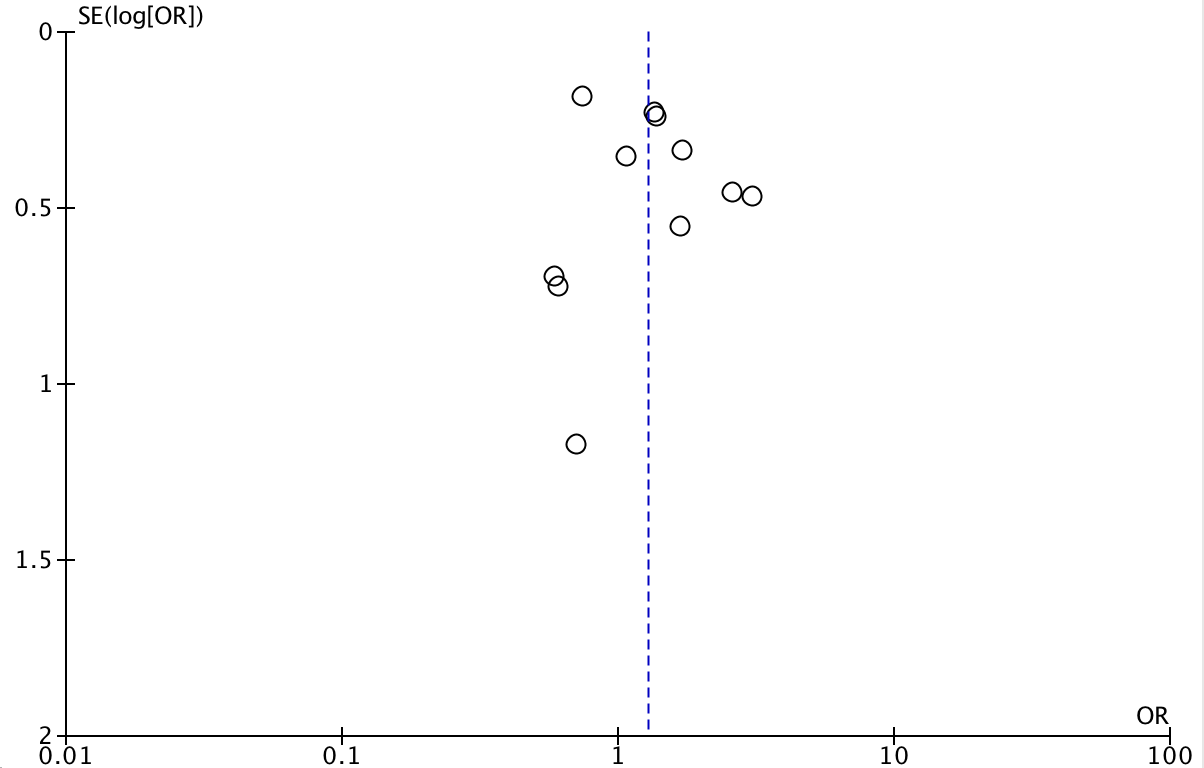


1. Cholangitis


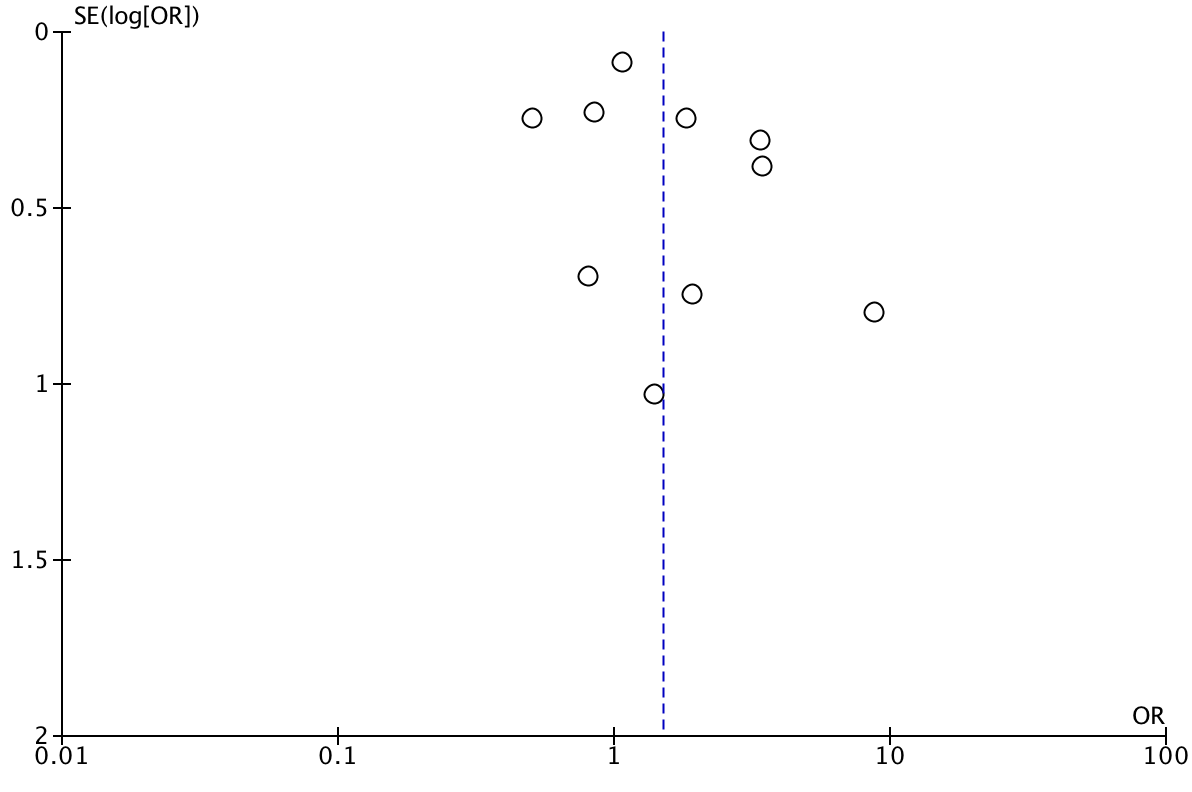

Supplement: gwae014_suppl_Supplementary_Tables [file gwae014_suppl_supplementary_tables.docx]
