# Supplementary figures and images for: Indication for endoscopic retrograde cholangiopancreatography and development of hemorrhage: a systematic review and meta-analysis
Source: J Can Assoc Gastroenterol. 2024 Apr 26;7(5):352–67. doi: 10.1093/jcag/gwae014 (PMC11477979; doi:10.1093/jcag/gwae014)

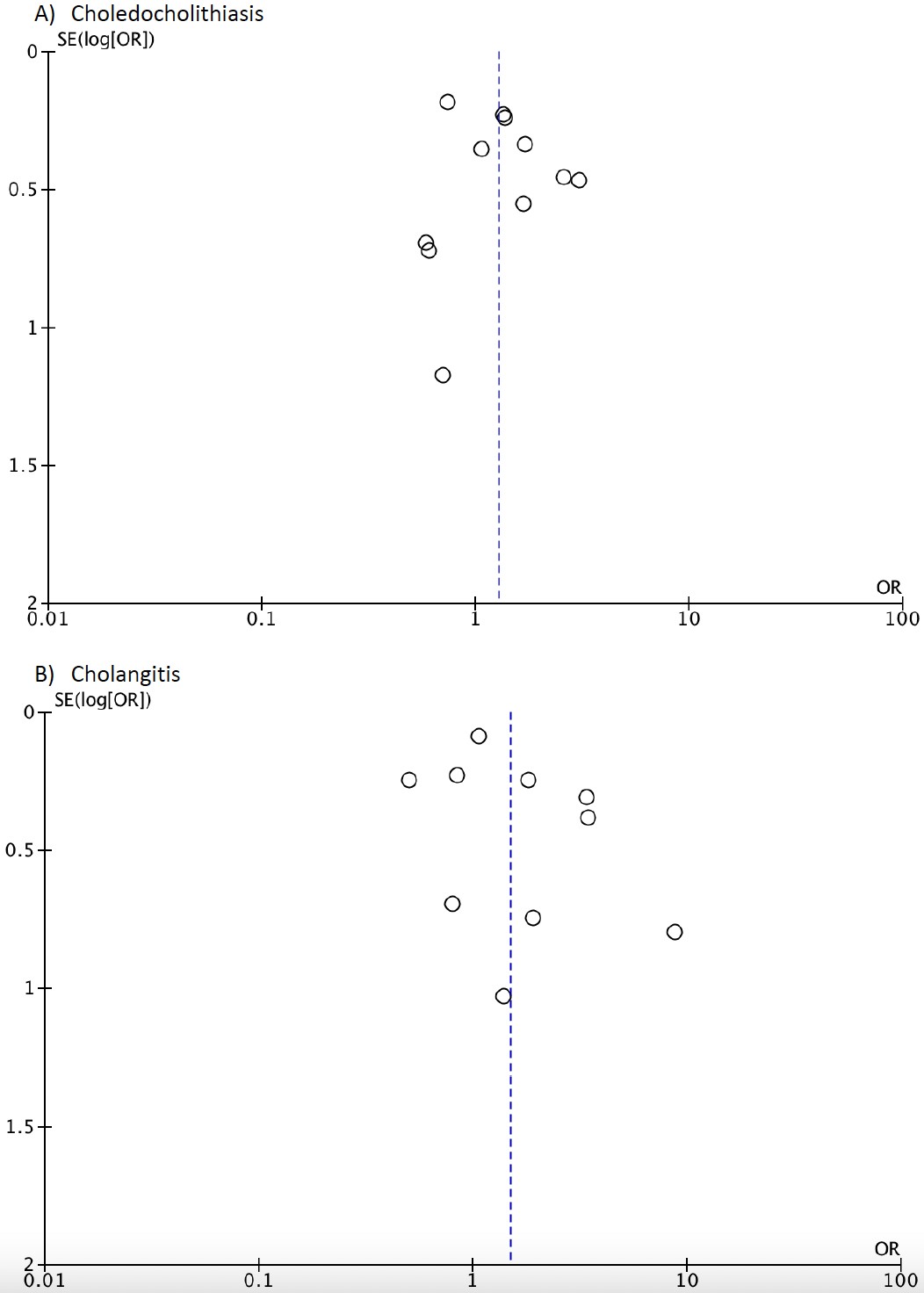

Supplement: gwae014_suppl_Supplementary_Figure_S1 [file gwae014_suppl_supplementary_figure_s1.jpeg]
